# Prefrontal Oxygenation in a Subjective Decision on a Situational Danger Assessment Task: Personality Traits and Decision-Making Styles Involvement

**DOI:** 10.3390/bs15050647

**Published:** 2025-05-09

**Authors:** Ferran Balada, Neus Aymamí, Óscar García, Luis F. García, Anton Aluja

**Affiliations:** 1 Lleida Institute for Biomedical Research, Dr. Pifarré Foundation, 25198 Lleida, Spain; naymami@gss.cat (N.A.); oscar.garcia@universidadeuropea.es (Ó.G.); luis.garcia@uam.es (L.F.G.); anton.aluja@udl.cat (A.A.); 2 Department of Psychobiology and Methodology of Health Sciences, Faculty of Psychology, Autonomous University of Barcelona, 08193 Barcelona, Spain; 3 Psychiatry, Mental Health and Addictions Service, Santa Maria Hospital of Lleida, 25198 Lleida, Spain; 4 Department of Psychology, European University of Madrid, 28670 Madrid, Spain; 5 Department of Biological and Health Psychology, Faculty of Psychology, Autonomous University of Madrid, 28049 Madrid, Spain; 6 Department of Psychology, Faculty of Psychology, University of Lleida, 25001 Lleida, Spain

**Keywords:** personality traits, prefrontal cortex, risk assessment, decision-making styles, fNIRS

## Abstract

This study investigated prefrontal cortex activity during the viewing and evaluation of pictures depicting scenarios with varying levels of danger, with a focus on the modulatory effects of personality traits and decision-making styles. The study sample included 120 male participants (44.4 ± 12.9 years) and 87 female participants (38.9 ± 10.5 years). Functional Near-Infrared Spectroscopy (fNIRS) was used to measure prefrontal oxygenation during the period of looking at pictures and the subsequent period of judging how dangerous they looked. Psychometric assessments included the Zuckerman–Kuhlman–Aluja Personality Questionnaire (ZKA-PQ) and the Melbourne Decision-Making Questionnaire (MDMQ). The results revealed significant time-by-region (*F* = 2.9, *p* = 0.013) and danger level by region interactions (*F* = 2.8, *p* = 0.021) during the viewing period. During the evaluation period, a significant time-by-region interaction was observed (*F* = 8.7, *p* < 0.001). High sensation seekers exhibited reduced oxygenation levels in specific right prefrontal regions, reflecting a differential neural response to varying danger levels. Similarly, individuals with higher Aggressiveness and Extraversion displayed distinct oxygenation patterns during the evaluation phase, suggesting that personality traits influence prefrontal activity. However, no significant effects of decision-making styles were detected in either phase. These findings emphasise the pivotal role of the prefrontal cortex in assessing scene safety and highlight how neural responses are modulated by personality traits, rather than by decision-making styles.

## 1. Introduction

Multiple studies have highlighted the implication of the prefrontal cortex in decision-making processes (Volz et al., 2006; Rosenbloom et al., 2012; Domenech & Koechlin, 2015). Various regions within the prefrontal cortex have been linked to specific aspects of decision-making and cognitive control (Coutlee & Huettel, 2012). The medial regions of the prefrontal cortex have been extensively studied (Volz et al., 2006; Bechara & Van Der Linden, 2005; Fellows, 2007; Martinez-Selva et al., 2006). However, other studies have shown the involvement of lateral areas, in addition to medial areas, in decision-making (Domenech & Koechlin, 2015; Ouerchefani et al., 2017), particularly in risk-related decision-making (Fecteau et al., 2007; Sela et al., 2012; Yang et al., 2017; Panidi et al., 2022), and risk-taking behaviours (Blankenstein et al., 2018).

Several studies have demonstrated changes in personality and executive functions in individuals with dorsolateral prefrontal cortex lesions (Forbes et al., 2014; Barrash et al., 2022). Several studies have shown differences in the activation of lateral areas based on personality traits (Rodrigo et al., 2016b; Wang et al., 2018; Balada et al., 2019). For example, high neuroticism and low sensation seeking scores have been lined to reduced prefrontal activity when viewing unpleasant pictures (Balada et al., 2019), whereas other studies indicate a positive relationship between neuroticism and oxygenation in lateral areas when exposed to neutral or happy faces (Lucas et al., 2019). In fact, patients with lesions in the left dorsolateral prefrontal cortex have higher neuroticism scores and lower conscientious scores (Forbes et al., 2014). In addition, introverts exhibit stronger emotional responses after stimulation of the dorsolateral area compared to extroverts (Peña-Gómez et al., 2011).

In a recent study, Acconito et al. (2023) emphasise the paucity of research addressing the neuroscientific underpinnings of decision-making styles. The authors propose that these styles are closely associated with physiological processes underlying the capacity to define and prioritise objectives, adapt to circumstantial changes, manage and assume risks, and tolerate and regulate stress. Several brain structures have been implicated in these processes (Szczepanski & Knight, 2014).

Previous findings have highlighted the role of the medial prefrontal cortex in functions such as self-representation of goals and risk-taking and its regulation (Klein-Flügge et al., 2022; Li et al., 2019; Orsini et al., 2018). Additionally, the lateral prefrontal cortex, particularly the dorsolateral region, has been associated with risk-taking and its management, adaptability to dynamic circumstances, and the regulation of emotions (Morawetz et al., 2019; Turnbull et al., 2019).

Jamali et al. (2019) investigated the neural response of the dorsolateral prefrontal cortex during subjective decision-making processes. They examined the subjective safety perceptions triggered by different visual stimuli of the same scenario. Intraneuronal recordings indicated a gradual change in neuronal activity, reaching equilibrium at a transitional point. These findings demonstrate the variability of neuronal activity in the dorsolateral prefrontal cortex during subjective decision-making. Disruption of this region leads to a loss of distinction between conflicting subjective decisions.

Furthermore, personality traits have been associated with decision-making styles, with neuroticism (positive) and extraversion (negative) linked to non-vigilant decision-making, and aggressiveness and activity are (positive) associated with a vigilant style (Urieta et al., 2021). Similarly, extroverts tend to exhibit a less rational decision-making style, while agreeableness and conscientiousness are associated with a rational style (El Othman et al., 2020). A recent study (Aluja et al., 2024), shows that the vigilant style is related with rational decision-making and the non-vigilant style with avoidance of decision-making. Therefore, the involvement of the prefrontal cortex in decision-making seems to be evident. Likewise, different personality traits, such as neuroticism or extraversion, seem to be related to decision-making styles and to the activity of the prefrontal cortex.

Despite existing evidence linking the prefrontal cortex to decision-making and personality traits, the interaction between personality traits, decision-making styles, and prefrontal activity remains underexplored. This exploratory study aims to investigate these dynamics using fNIRS (Functional Near-Infrared Spectroscopy) by examining changes in prefrontal oxygenation during subjective decision-making tasks involving visual stimuli. Specifically, we will examine how personality traits such as neuroticism, extraversion, and sensation seeking, as well as decision-making styles (e.g., vigilant vs. non-vigilant), influence prefrontal activation, particularly in the dorsolateral prefrontal cortex. Despite the exploratory nature of this study, findings from Jamali et al. (2019) suggest that increased activity in the dorsolateral prefrontal areas is to be expected in situations where participants demonstrate elevated levels of hesitation when determining whether a given picture represents a safe or unsafe scenario. Furthermore, greater activation of the dorsolateral prefrontal cortex is expected to be linked to a vigilant decision-making style, and its associated personality traits, as it enhances information integration and thorough evaluation of choices. By integrating behavioural and neurophysiological measures, this research aims to provide novel insights into how individual differences and decision-making styles shape neural activity during decision-making processes, extending the existing literature and advancing our understanding of the relationship between personality, decision-making styles, and prefrontal function.

## 2. Material and Methods

### 2.1. Participants

The participants in this study were healthy volunteers who responded to a recruitment notice disseminated via the university’s institutional email and advertisements placed in the local press. Prior to participation, they were fully informed about the experimental procedures and provided written informed consent. All participants were instructed to abstain from smoking and consuming stimulants, including coffee and tea, on the day of the experiment. The final sample comprised 120 men (mean age 44.4 ± 12.9 years) and 87 women (mean age 38.9 ± 10.5 years), all of whom received a €25 honorarium for their participation. The experimental protocol was approved by the Ethics Committee of the University of Lleida and Arnau de Vilanova University Hospital University.

### 2.2. Procedure

Experimental recordings were conducted in an electromagnetically and acoustically isolated Faraday cage, which contained separate compartments for the researcher and the participant. Visual stimuli were presented on a 32-inch television screen, with participants seated in an ergonomic chair. Once the signal recording sensor was in place, the mechanics of the task to be performed were explained to the participant and any doubts were clarified. Prior to stimulus presentation, a 10 min baseline recording was made, followed by the behavioural task. The total task duration was 12 min and 15 s. After the fNIR recording, participants completed a computerised version of the questionnaires.

### 2.3. Behavioural Task: Subjective Situational Assessment

**Figure 1:**
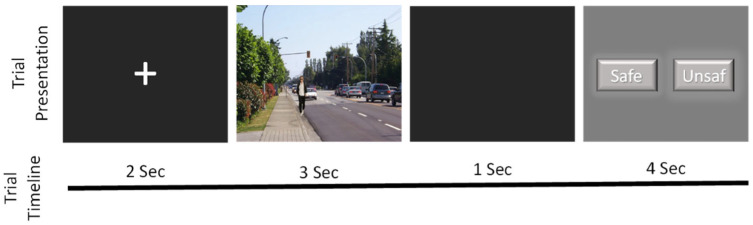
Design of the experimental procedure for the presentation of the pictures. There was an inter-trial interval of 10 s between the presentation of one picture and the next.

The task utilised a set of 35 pictures, provided by Dr. Ziv Williams and previously validated in earlier studies (Jamali et al., 2019). These pictures were grouped into seven sets, each containing five pictures, depicting a person interacting with an object in various positions that altered the perceived risk of the situation. Participants were required to evaluate whether each situation appeared dangerous or safe. The pictures were presented in a randomised order. Figure 1 shows the presentation design. Each trial started with a fixation stimulus displayed for 2 s, followed by the presentation of an image for 3 s (viewing period). After a delay of 1 s, a rating screen was shown to the participant for 4 s, where they assessed if the picture represents a dangerous situation (evaluation period). This procedure was repeated for all 35 pictures, with a 10 s inter-trial interval between each presentation. The response screen remained visible for 4 s, with the positions of the safe or unsafe buttons alternating randomly.

### 2.4. Functional Near-Infrared Spectroscopy (fNIR) Recording

Prefrontal cortex activity was recorded using the fNIR 1100 system (fNIR Devices LLC, Potomac, MD, USA; Biopac Systems Inc., Goleta, CA, USA). This system detects changes in oxygenated (HbO) and deoxygenated (Hbr) haemoglobin levels by measuring the response to 730 nm and 850 nm wavelength light. The fNIR 1100 system (Biopac Systems, Inc.) uses a fixed, predefined probe layout optimised for measuring haemodynamic responses in the prefrontal cortex. The fNIR 1100 probe features a rectangular matrix of optical components with four emitters and ten detectors arranged to create 16 functional channels. The interoptode distance (distance between emitters and detectors) is approximately 2.5–3 cm. The frontal sensor was positioned on the supraorbital prefrontal regions F7, Fp1, Fpz, Fp2 and F8 according to the International 10–20 EEG system, corresponding to Brodmann areas 10, 11, 45, 46 and 47 (Rodrigo et al., 2016a). The main aspects of the fNIR setup, placement of the fNIR sensor-pad, acquisition, processing and analysis of the fNIR data are described in Ayaz et al. (2011).

The signal was processed using COBI data collection suite (Biopac System Inc., Goleta, CA, USA), which controls light intensity and signal amplification, as well as synchronises with picture stimuli and participant responses recorded by E-Prime 3.0 software (Psychology Software Tools, Inc., 2016). The acquired signal was filtered using a low-pass finite impulse response (FIR) filter with a cut-off frequency of 0.1 Hz and an order of 20. The Sliding-window Motion Artefact Rejection (SMAR) algorithm was then applied to remove motion artefacts and exclude channels with signal saturation or insufficient impulse intensity (400–4000 mV). Hemodynamic data, calculated using the modified Beer–Lambert law, were baseline-corrected to allow for comparison across stimuli. These data were then further refined through linear trend reduction and constrained signal deviation. For conventional averaging analysis, the filtered and pre-processed haemodynamic signals were segmented into epochs centred on each stimulus onset. Each epoch ranged from 2 s before stimulus onset (baseline period) to 9 s after stimulus onset (response period). The baseline period was used to normalise each epoch by subtracting the mean pre-stimulus signal. Hemodynamic responses from all repetitions of the same stimulus condition were then averaged for each channel to generate a representative response curve for each condition. Mean values were calculated for each second, and hemodynamic measures were grouped into eight regions—left dorsolateral (channels 1, 3), left ventrolateral (channels 2, 4), left dorsorostral (channels 5, 7), left ventrorostral (channels 6, 8), right dorsorostral (channels 9, 11), right ventrorostral (channels 10, 12), right dorsolateral (channels 11, 13), and right ventrolateral (channels 14, 16)—based on LED (light-emitting diode) source channels.

### 2.5. Psychometric Measures

#### 2.5.1. Zuckerman–Kuhlman–Aluja Personality Questionnaire Shortened Form

The shortened version of the Zuckerman–Kuhlman–Aluja Personality Questionnaire (ZKA-PQ/SF; Aluja et al., 2018), was used to assess five personality factors: Aggressiveness (AG), Activity (AC), Extraversion (EX), Neuroticism (NEU), and Sensation Seeking (SS). This version consists of 80 items, with four items per facet across 20 facets, rated on a four-point Likert scale ranging from totally disagree (1) to totally agree (4). The questionnaire has demonstrated high reliability and validity in psychometric and cross-cultural studies. For this sample, the reliability coefficients were 0.90 for Aggressiveness, 0.83 for Activity, 0.88 for Extraversion, 0.91 for Neuroticism, and 0.84 for Sensation Seeking (Table 1).

#### 2.5.2. Melbourne Decision-Making Questionnaire (MDMQ)

The Melbourne Decision-Making Questionnaire (MDMQ; Mann et al., 1997) was utilised to evaluate decision-making styles according to the Conflict Theory model. This 22-item questionnaire assesses four factors: Vigilance (VIG), Hypervigilance (HYPER), Buck-passing (BUCK-PAS), and Procrastination (PROCR), with responses rated on a three-point Likert scale (true, sometimes true, false). The version used in this study was validated by Alzate Saez De Heredia et al. (2004) and its psychometric properties were replicated by Urieta et al. (2022). For the present sample, the reliability coefficients were 0.77 for Vigilance, 0.79 for Hypervigilance, 0.82 for Buck-passing, and 0.82 for Procrastination (Table 1).

### 2.6. Data Analysis

All analyses were performed using SPSS software (version 27), with an alpha level of 0.05 for significance. Effect sizes were reported as partial eta squared (*η_p_*^2^). Mean and standard deviations were calculated for ZKA-PQ factors and MDMQ styles for each gender and *t*-test comparison was made. Cronbach’s alphas were calculated for each scale. Pearson correlation coefficients were calculated to evaluate the relationships between personality factors and decision-making styles. Reaction time was analysed using a general linear model (GLM) with repeated measures, considering a full factorial design. The model included the reaction time measures for each danger level as a within-subject factor. A type III sum of squares was used. Statistical significance of effects was assessed using an F-test, and post hoc pairwise comparisons were performed using the Bonferroni correction to control for type I error. Sphericity assumptions were tested using Mauchly’s test and, if violated, Greenhouse–Geisser corrections were applied to adjust for degrees of freedom. Separate repeated measures ANOVA were performed to evaluate the effect of time, danger level, gender and prefrontal areas on mean oxygenation level during viewing or evaluation of pictures. The Mauchly test indicated that the assumption of sphericity was violated for all independent variables in the analysis during the visualisation period, whereas the danger level met the assumption of sphericity during the evaluation period. Degrees of freedom were corrected using Greenhouse–Geiser estimates of sphericity. To examine the effect of personality variables and decision-making styles, we divided each of these variables into three groups according to the 33rd and 66th percentiles. We then performed a separated repeated measures analysis, of both viewing time and picture rating time, using time, danger level and prefrontal region as within-group factors, and personality variable or decision-making style as a between-group variable.

## 3. Results

**Figure 2:**
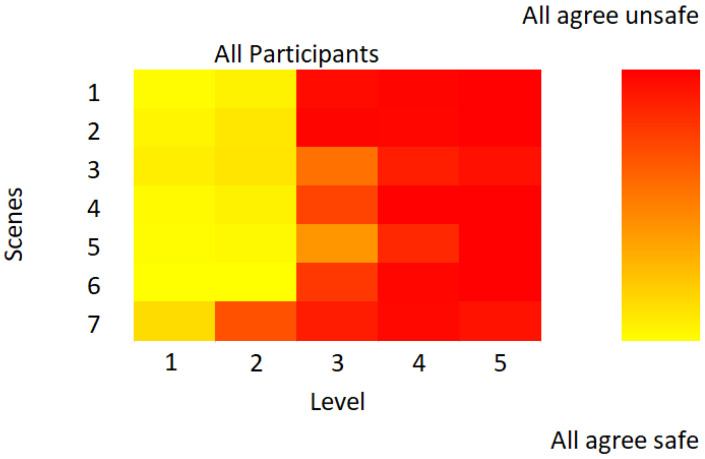
Percentage of participants who thought that the picture shown could be a sign of a dangerous situation. The percentage of responses is indicated by a color gradation: yellow (indicates safety) to red (safety considered unsafe).

There were no significant differences between genders in the total number of pictures rated as safe (*F* = 1.04; *p* = 0.06), nor was there a significant correlation of this variable with age (r = −0.05; *p* = 0.49). Figure 2 shows the percentage of respondents who rated a picture as safe as a function of scene and level of danger. The percentages corresponding to the responses indicated as safe situations for the five levels evaluated were 95.9%, 85.6%, 24.1%, 5.5% and 2.4%, respectively, while the mean values for the different scenes ranged from 24.4% (scene 7) to 46.1% (scene 5). An analysis of reaction time as a function of danger level revealed significant differences (*F*_(3.5, 708)_ = 13.3, *p* < 0.001, *η_p_*^2^ = 0.06), with a quadratic fit (*F*_(1, 204)_ = 34.4, *p* < 0.001, *η_p_*^2^ = 0.14). Post hoc pairwise comparisons indicated that the highest reaction time occurred at level 3 (1318.12 ± 21.8 ms), which was significantly different from level 1 (*p* = 0.003), level 4 (*p* < 0.001) and level 5 (*p* < 0.001). In contrast, the lowest reaction time was observed at level 5 (1196.1 ± 18.8 ms), showing significant differences from level 1 (*p* = 0.01), level 2 (*p* < 0.001), and level 3 (*p* < 0.001).

Table 1 shows the means and standard deviations of the Zuckerman–Kuhlman–Aluja Personality Questionnaire (ZKA-PQ) and Melbourne Decision-Making Questionnaire (MDMQ) scales for each sex. Additionally, the table includes sex-based differences, internal consistency values for each scale, and correlations between these scales. All scales demonstrated substantial internal consistency, with Cronbach’s alpha values ranging from 0.83 for the Activity scale to 0.91 for the Neuroticism scale in the ZKA-PQ. The MDMQ scales obtained an internal consistency ranging from 0.77 for the Vigilance scale to 0.82 for the Buck Passing and Procrastination scales.

### 3.1. Prefrontal Oxygenation

The examination of repeated measures of oxygenation levels across prefrontal regions during picture viewing, taking into account the level of danger associated with the pictures and the gender of the participants, revealed interactions between viewing time and prefrontal region (*F*_(4.3, 860.4)_ = 2.9, *p* = 0.013, *η_p_*^2^ = 0.01), as well as between viewing time and danger level (*F*_(4.4, 864.4)_ = 2.8, *p* = 0.021, *η_p_*^2^ = 0.01). Moreover, a notable main effect of the prefrontal region was detected (*F*_(4.3, 860.4)_ = 7.0, *p* < 0.001, *η_p_*^2^ = 0.03). No remarkable differences related with gender were found (*F*_(1, 198)_ = 0.7, *p* = 0.40). Post hoc comparisons demonstrated lower oxygenation levels in the ventrolateral compared to the ventrorostral areas in both hemispheres (left *p* < 0.001; right *p* = 0.03).

**Figure 3:**
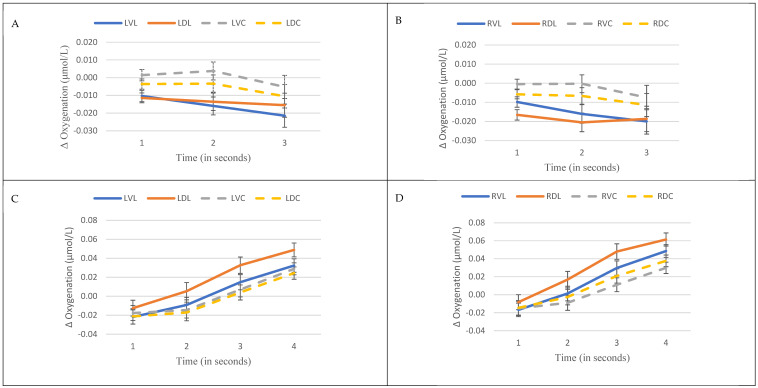
Oxygenation levels in different prefrontal areas over time. (**A**) Mean oxygenation in the left hemisphere during picture viewing; (**B**) Mean oxygenation in the right hemisphere during picture viewing; (**C**) Mean oxygenation in the left hemisphere during picture evaluation; (**D**) Mean oxygenation in the right hemisphere during picture evaluation).

Figure 3 presents the temporal trends of oxygenation in the left (Figure 3A) and right (Figure 3B) prefrontal regions. Rostral areas, particularly ventrorostral, exhibit the highest oxygenation levels. Additionally, lateral areas exhibit a rapid decrease in oxygenation between the first and second seconds, whereas this decrease was not observed in the rostral areas.

The most pronounced effects were observed during the picture evaluation period, with significant interactions between time and prefrontal region (*F*_(5.4, 1077.2)_ = 8.7, *p* < 0.001, *η_p_*^2^ = 0.04), as well as main effects for prefrontal region (*F*_(5, 991.3)_ = 8.2, *p* < 0.001, *η_p_*^2^ = 0.04), time (*F*_(1.3, 260.9)_ = 71.1, *p* < 0.001, *η_p_*^2^ = 0.26) and danger level (*F*_(3.9, 772.1)_ = 3.4, *p* = 0.01, *η_p_*^2^ = 0.02). Again, no significant effects were found for gender during this period (*F*_(1, 198)_ = 1.2, *p* = 0.27). Post hoc analysis revealed a significant increase in oxygenation levels during this period, with oxygenation levels increasing sequentially for each time point (all *p* < 0.001). Pairwise analysis for danger levels indicate that the most dangerous level showed lower oxygenation levels than the third (*p* = 0.005) and the four (*p* = 0.02) danger levels. Moreover, the highest oxygenation levels were observed in dorsolateral regions. In the left hemisphere, the dorsolateral area exhibit higher oxygenation levels compared to other left regions (ventrolateral *p* = 0.02; dorsorostral *p* = 0.03; ventrorostral *p* = 0.06). In the right hemisphere, the dorsolateral area displayed higher oxygenation levels compared to ventrorostral (*p* < 0.001) and dorsorostral (*p* = 0.004) regions. The lateral regions, particularly dorsolateral areas, exhibited the highest mean oxygenation levels. Figure 3 illustrates the temporal oxygenation patterns in the left (Figure 3C) and right (Figure 3D) hemispheres with the most significant increases observed in the dorsolateral regions.

**Figure 4:**
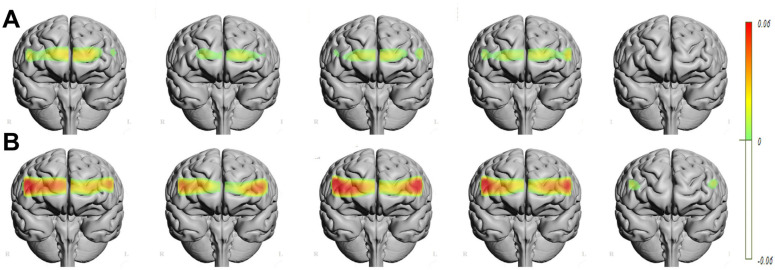
Representation of hemodynamic changes in the prefrontal cortex as a function of the danger level of the pictures (columns from left to right correspond to levels 1 through 5). The top row (**A**) illustrates the analysis during the picture-viewing period, where repeated measures analysis revealed significant differences in the right lateral areas (all *p* < 0.05) and a trend toward significance in the left ventral areas (all *p* < 0.07). The bottom row (**B**) depicts the analysis during the picture evaluation period, where repeated measures analysis demonstrated significant differences across all prefrontal areas (all *p* < 0.04) except the left dorsolateral area (*p* = 0.15) (Topograph 4.11© Frontal View. Interpolated Bordered. Thr. 128).

Figure 4A illustrated changes in oxygenation levels across different prefrontal areas during picture viewing, while Figure 4B captures oxygenation patterns during picture evaluation.

### 3.2. Subgroup Analysis Based on ZKA-PQ and MDMQ Scores

The gender variable was excluded from the analysis of the scores obtained in the questionnaires due to an absence of significant differences in the results corresponding to prefrontal oxygenation. Table 2 demonstrates the repeated measures analysis conducted during picture viewing, which was stratified based on subgroups according to ZKA-PQ and MDMQ scores. The most significant finding was a strong interaction observed between danger level, time, prefrontal areas, and the Sensation Seeking scale (Wilk’s *λ* = 0.42, *F*_(112, 284)_ = 1.4, *p* = 0.018, *η_p_*^2^ = 0.35). Further post hoc analysis revealed significant differences in oxygenation levels between groups in the areas of the right hemisphere, particularly in the ventrorostral (*F*_(2, 200)_ = 4.9, *p* = 0.009, *η_p_*^2^ = 0.05) and dorsolateral (*F*_(2, 200)_ = 6.0, *p* = 0.003, *η_p_*^2^ = 0.06) regions. High sensation seekers displayed lower oxygenation levels compared to low and medium sensation seekers in these areas (all *p* < 0.03). Lower sensation seekers experienced an increase in oxygenation when exposed to low danger pictures (levels 1, 2, and 3) and a decrease in oxygenation when viewing high danger pictures (levels 4 and 5). Conversely, higher sensation seekers displayed a decrease in oxygenation when viewing low danger pictures and either maintained or increased their oxygenation when exposed to high danger pictures. One-way ANOVA results also indicated a significant effect for the right dorsolateral (*F*_(2, 200)_ = 6.1, *p* = 0.003, *η_p_*^2^ = 0.06); ventrorostral (*F*_(2, 200)_ = 4.9, *p* = 0.009, *η_p_*^2^ = 0.05); and dorsorostral (*F*_(2, 201)_ = 3.2, *p* = 0.042, *η_p_*^2^ = 0.03) and a trend for the right ventrolateral (*F*_(2, 200)_ = 2.8, *p* = 0.064) areas, with higher oxygenation levels in ventrorostral region for participants with low (*p* = 0.027), and medium (*p* = 0.017) scores than participants with highest scores in sensation seeking.

Table 3 presents the repeated measures analysis for the evaluation period, highlighting significant interactions among all variables for the ZKA-PQ Aggressiveness (Wilk’s *λ* = 0.26, *F*_(168, 228)_ = 1.3, *p* = 0.023, *η_p_*^2^ = 0.49) and Extraversion (Wilk’s *λ* = 0.26, *F*_(168, 228)_ = 1.3, *p* = 0.025, *η_p_*^2^ = 0.49) scales. Separate analysis for each area revealed a significant interaction between aggressiveness groups, time, and danger level in the right lateral areas, particularly the ventral (Wilk’s *λ* = 0.81, *F*_(24, 378)_ = 1.7, *p* = 0.02, *η_p_*^2^ = 0.10) and dorsal (Wilk’s *λ* = 0.81, *F*_(24, 378)_ = 1.7, *p* = 0.026, *η_p_*^2^ = 0.10) regions, as well as in the left ventrolateral area (Wilk’s *λ* = 0.83, *F*_(24, 378)_ = 1.6, *p* = 0.047, *η_p_*^2^ = 0.09). Additionally, a trend towards significance was observed for the left dorsolateral (Wilk’s *λ* = 0.84, *F*_(24, 376)_ = 1.4, *p* = 0.08) and dorsorostral (Wilk’s *λ* = 0.84, *F*_(24, 380)_ = 1.4, *p* = 0.08) regions. On the other hand, male participants with high aggressiveness scores had greater right dorsolateral activity (*p* = 0.039) than those with low aggressiveness scores.

In the individual analyses for each region, no significant effects were observed for the trait of extraversion. There was only a tendency towards significance for women with higher extraversion scores to have greater activity in the right ventrolateral areas (*p* = 0.087).

In the analysis of decision-making styles, only the existence of an interaction depending on the area studied was observed for the procrastination style. A more detailed study shows the existence of significant differences between individuals with higher (−0.01 ± 0.20) scores on this variable and those with medium (0.06 ± 0.17) and lower (0.05 ± 0.17) scores in the right dorsolateral area (*F*_(2, 200)_ = 3.32, *p* = 0.038).

## 4. Discussion

The primary findings of this study indicate the activation of distinct regions within the prefrontal cortex during the observation of pictures varying in levels of danger and their subsequent evaluation. Greater activation was observed in the rostral prefrontal cortex during picture viewing compared to the lateral prefrontal regions. Conversely, during the evaluation period, maximal prefrontal activity occurred in the lateral regions, particularly within the dorsolateral areas. This prefrontal response appears to be modulated by personality traits. Decision-making styles, however, did not exhibit a significant impact. It is important to note that the scales used in this study demonstrated high internal consistency, as well as relationships between the personality and decision-making scales that align with the findings of Urieta et al. (2021), conducted on a large sample of over 1500 participants. Furthermore, the longer reaction times observed for images corresponding to the third level of danger suggest that decision-making difficulty is greatest at this level.

Jamali et al. (2019), using the same pictures, reported changes in intraneuronal activity, identifying an equipoise point that reflects shifting decisions. This equilibrium zone is associated with subjective decision-making rather than sensory or motor choices. In the current study, minor changes were observed in the ventrorostral or central prefrontal cortex during the perceptual period. However, during the evaluation period, dorsolateral prefrontal cortex activation was highest, particularly at intermediate danger levels, where participants assessed safe or unsafe, leading to longer reaction times. Jamali et al. (2019), indicated that peak neural activity is detected at the equipoise point. These findings align with prior studies linking dorsolateral prefrontal activity to risk-related decision-making across various methods. Several studies have shown that dorsolateral prefrontal modulation is associated with decision-making in high-risk scenarios (Yang et al., 2017; Panidi et al., 2022; Ye et al., 2015; Khaleghi et al., 2020), with a particular emphasis on the right dorsolateral prefrontal region. Blankenstein et al. (2018) found an inverse relationship between dorsolateral activity and real-life risk reporting, implicating the dorsolateral prefrontal area cortex in integrating cognitive and emotional cues during decision-making. Other research suggests that of lateral prefrontal activation, which regulates emotional responses, has a significant impact risk-related decision-making (Turnbull et al., 2019). Studies using electroencephalography further support these findings, showing that enhanced electrical activity in the right prefrontal cortex correlates with increased risk aversion (Gianotti et al., 2009), highlighting the role of the dorsolateral prefrontal cortex in risky decision-making. These results and those of Jamali et al. (2019), suggest that this area activates mainly during subjective evaluation of safety or risk.

This study also revealed subtle differences in ventrorostral prefrontal areas during picture viewing. Other studies have demonstrated that the ventromedial prefrontal cortex connects strongly with reward-related regions implicated in risk-taking behaviour (van Holstein & Floresco, 2020; Rolls et al., 2022), suggesting that reward-processing areas may impact risk-related behaviours. Research shows lower levels of oxygenation in the right prefrontal cortex of high sensation seekers, whereas no significant differences were observed in the left hemisphere. This aligns with findings from risk decision studies that suggest that risk decision-making is lateralised in the right hemisphere (Khaleghi et al., 2020). Additionally, reduced theta rhythm (indicative of lower activity) in the right prefrontal cortex has been identified as a strong predictor of risky choices (Studer et al., 2013). The decreased activity observed in this hemisphere among high sensation-seekers suggests an increased propensity for risk-taking behaviour, possibly due to the appetitive nature of certain risky activities for these individuals. Moreover, differences in brain connectivity, such as the ventral attentional and frontoparietal networks, have been associated with risk propensity (Baltruschat et al., 2019). Structural changes have also been linked to sensation seeking (Holmes et al., 2016). The task employed in this study, which involved judging levels of danger represented in pictures, may explain the observed relationships between prefrontal activity and the sensation seeking trait. A task involving greater emotional engagement might have elicited associations with other dimensions more related to emotions. However, it is important to acknowledge the spatial limitations of fNIRS, which may restrict detection of activity in medial prefrontal areas, potentially impacting effect size (Taylor et al., 2020). Furthermore, the categorisation of variables may have affected the sensitivity of the analysis, thereby reducing statistical power (DeCoster et al., 2009).

We also observed that personality traits, specifically aggressiveness and extraversion, modulated prefrontal activity during picture evaluation. Several studies have shown links between personality traits and prefrontal cortex activity (Rodrigo et al., 2016b; Wang et al., 2018; Balada et al., 2019; Lucas et al., 2019). Transcranial magnetic stimulation of the dorsolateral cortex has been shown to reduce self-reported aggressiveness in prisoners (Molero-Chamizo et al., 2019), and proactive aggression correlates with greater grey matter in the dorsolateral prefrontal cortex and reduced connectivity between the left posterior cingulate cortex and the right dorsolateral prefrontal area (Zhu et al., 2019). Hemispheric asymmetry in alpha electroencephalographic activity, with right hemisphere dominance has been suggested as a marker of aggressive behaviour (Keune et al., 2012). These data suggest that levels of aggression-related personality traits modulate lateral prefrontal activity, particularly in dorsolateral areas. However, most of these studies examining this asymmetry have done so in resting conditions. Other research has implicated prefrontal asymmetry in motivational mechanisms, linking right hemisphere activation with withdrawal and left activation with approach behaviours (Harmon-Jones & Gable, 2018). In our study, right prefrontal activation was more pronounced in participants with higher Aggressiveness scores, potentially reflecting greater sensitivity to dangerous situations, as this trait correlates with neuroticism, procrastination and hypervigilance. On the other hand, it is remarkable that the hemispheric lateralisation observed in relation to personality traits is not observed when analysing the response of the prefrontal cortex during task performance. Other studies have shown the involvement of the left prefrontal cortex in decision-making in perceptual (Heekeren et al., 2004) or semantic (Simmons et al., 2005) tasks.

A notable aspect was the absence of significant relationships between decision-making styles, as assessed by the MDMQ, and prefrontal activity during the decision-making task. Several factors may explain these null results. First, the MDMQ is designed to assess decision-making under stressful situations (Mann et al., 1997), whereas the present study involved a task focusing on subjective decision-making. Indeed, the personality dimensions most strongly associated with the MDMQ scales, neuroticism and extraversion, are predominantly related to emotions (Barańczuk, 2019), and neither demonstrated significant associations with prefrontal oxygenation in this study. However, these results cannot be taken as proof of the non-existence of the effects. Future research should incorporate modifications of the experimental context, such as inclusion of emotional or stress-inducing scenarios, to better assess decision-making processes under different conditions.

Although this study has several strengths, certain limitations must be noted. The fNIRS technique captures activity only in superficial cortical areas, limiting its application for deeper structures like medial prefrontal and orbitofrontal cortex or limbic regions. Additionally, using static pictures and a single decision task may constrain generalizability of the findings. It is important to acknowledge that, despite the analysis being conducted across two distinct periods during the study, there is inherent overlap between the neural responses associated with image perception and those related to decision-making. Notably, the decision-making process begins concurrently with the presentation of the images. However, neural responses to low-complexity tasks, such as image perception, are typically faster than those elicited by high-complexity tasks, such as decision-making. This overlap should be considered when interpreting the haemodynamic response dynamics observed in our study.

Future studies should aim to address the limitations of the present study and explore unresolved questions, such as the near absence of a relationship between decision-making styles and prefrontal activity. To achieve this, it will be crucial to use methodologies that allow for the assessment of deeper structures potentially involved in these processes and to employ experimental designs capable of evaluating responses across varied decision-making scenarios.

In conclusion, our results highlight variations in prefrontal activity during the perceptual process of picture presentation and their subsequent evaluation. Particularly, significant dorsolateral prefrontal activation during the decision period was observed. Personality traits influence cortical responses across both periods, with sensation-seeking affecting perception and aggressiveness and extraversion influencing decision-making. Decision-making styles, however, did not show notable effects.

## Figures and Tables

**Table 1:** Mean and standard deviation of men and women for the variables in the questionnaires. Difference between both sexes, Cronbach’s *α* and Pearson’s correlations.

	Men		Women					Procrastination	Buck-Passing	Hypervigilance	Vigilance	Sensation Seeking	Neuroticism	Extraversion	Activity
	Mean	Sd	Mean	Sd	*t*	*p* =	*α*								
Aggressiveness	31.8	9.3	31.1	8.4	0.57	0.57	0.90	**0.24**	0.08	0.21	−0.15	**0.27**	**0.38**	−0.09	0.02
Activity	42.0	7.8	43.2	6.9	−1.2	0.23	0.83	−0.11	−0.15	0.05	0.18	0.21	0.10	0.18	
Extraversion	47.5	7.7	50.2	8.3	−2.4	0.018	0.88	**−0.34**	**−0.34**	**−0.28**	0.1	0.19	**−0.30**		
Neuroticism	33.5	9.1	35.9	9.8	−1.8	0.081	0.91	**0.55**	**0.47**	**0.66**	−0.13	0.02			
Sensation Seeking	38.2	8.0	35.9	7.9	2.0	0.043	0.84	0.03	−0.13	−0.13	−0.10				
Vigilance	9.6	2.2	10.2	1.9	−2.1	0.039	0.77	−0.16	0.21	0.04					
Hypervigilance	4.1	2.3	5.3	2.5	−3.4	**0.001**	0.79	**0.51**	**0.59**						
Buck-Passing	4.5	2.5	5.0	2.5	−1.3	0.19	0.82	**0.61**							
Procrastination	3.1	2.3	3.1	2.4	−0.0	1.0	0.82								

Note. Differences between both sexes and correlations that are significant after Bonferroni correction for multiple comparisons are shown in bold.

**Table 2:** Repeated measures analysis conducted during picture viewing, which was stratified based on subgroups according to ZKA-PQ and MDMQ scores.

Effect	ZKA-PQ										MDMQ							
	AG		AC		EX		NEU		SS		VIG		HYPER		BUCK-PAS		PROCR	
	*F*	*p* <	*F*	*p* <	*F*	*p* <	*F*	*p* <	*F*	*p* <	*F*	*p* <	*F*	*p* <	*F*	*p* <	*F*	*p* <
Time	2.3	0.059	0.88	0.47	0.85	0.50	1.86	0.12	1.48	0.21	2.72	0.029	2.31	0.058	1.29	0.27	1.72	0.14
Level	1.43	0.18	0.74	0.65	0.8	0.60	1.32	0.23	**2.88**	**0.004**	0.69	0.70	0.86	0.55	0.39	0.93	0.46	0.88
Area	0.73	0.75	1.32	0.19	0.9	0.56	0.6	0.87	1.63	0.069	0.78	0.69	0.7	0.78	1.06	0.39	**1.76**	**0.042**
Level * Time	1.09	0.37	0.76	0.73	0.8	0.69	**1.84**	**0.025**	**1.83**	**0.026**	0.33	0.99	1.31	0.19	1.6	0.067	1.01	0.45
Time * Area	0.81	0.74	1.28	0.16	0.93	0.57	0.45	0.99	1.32	0.13	0.87	0.66	0.75	0.82	0.73	0.85	1.16	0.26
Level * Area	0.96	0.56	1.18	0.19	1.13	0.26	1.01	0.46	**1.38**	**0.045**	0.91	0.66	1.15	0.22	1.01	0.46	0.78	0.87
Level * Time * Area	1.09	0.28	1.07	0.32	1.06	0.35	1.02	0.44	**1.38**	**0.018**	0.96	0.60	1.11	0.24	1.01	0.48	1.14	0.19

Note. * = interactions between variables; *p* ≤ 0.05 and *η_p_*^2^ ≥ 0.06 highlighted in bold. ZKA-PQ = Zuckerman–Kuhlman–Aluja Personality Questionnaire; AG = Aggressiveness; AC = Activity; EX = Extraversion; NEU = Neuroticism; SS = Sensation Seeking; MDMQ = Melbourne Decision-Making Questionnaire; VIG = Vigilance; HYPER = Hypervigilance; BUCK-PAS = Buck-passing; PROCR = Procrastination.

**Table 3:** Repeated measures analysis conducted during picture evaluation, which was stratified based on subgroups according to ZKA-PQ and MDMQ scores.

Effect	ZKA-PQ										MDMQ							
	AG		AC		EX		NEU		SS		VIG		HYPER		BUCK-PAS		PROCR	
	*F*	*p* <	*F*	*p* <	*F*	*p* <	*F*	*p* <	*F*	*p* <	*F*	*p* <	*F*	*p* <	*F*	*p* <	*F*	*p* <
Time	2.22	0.041	0.66	0.68	1.88	0.084	1.22	0.30	1.41	0.21	1.76	0.11	1.49	0.18	0.37	0.90	0.73	0.62
Level	1.27	0.26	1.11	0.36	1.01	0.43	1.49	0.16	1.45	0.18	0.45	0.89	0.8	0.60	0.85	0.56	0.63	0.76
Area	0.63	0.84	1.49	0.11	0.78	0.69	0.56	0.89	1.57	0.086	1.29	0.21	0.78	0.69	0.47	0.95	0.91	0.55
Level * Time	1.42	0.09	0.89	0.61	0.35	1.0	1.35	0.13	1.21	0.23	0.61	0.93	0.73	0.82	0.75	0.80	1.08	0.37
Time * Area	0.99	0.50	0.91	0.64	0.86	0.73	1.08	0.34	0.95	0.56	1.02	0.44	0.88	0.69	0.71	0.91	0.76	0.86
Level * Area	1.31	0.08	1	0.48	1.03	0.42	1.06	0.36	1.14	0.25	1.02	0.45	1.05	0.39	0.94	0.61	1.32	0.072
Level * Time * Area	1.33	0.023	0.88	0.80	1.32	0.025	1.02	0.46	0.91	0.75	0.89	0.78	1.01	0.48	0.88	0.80	1.08	0.29

Note. * = interactions between variables.

## Data Availability

Full data will not be available until the project is completed. However, partial data can be requested from the first author.
